# Cost-Efficient Domain-Adaptive Pretraining of Language Models for Optoelectronics Applications

**DOI:** 10.1021/acs.jcim.4c02029

**Published:** 2025-02-11

**Authors:** Dingyun Huang, Jacqueline M. Cole

**Affiliations:** † Cavendish Laboratory, Department of Physics, University of Cambridge, J. J. Thomson Avenue, Cambridge CB3 0HE, U.K.; ‡ ISIS Neutron and Muon Source, Rutherford Appleton Laboratory, Harwell Science and Innovation Campus, Didcot, Oxfordshire OX11 0QX, U.K.

## Abstract

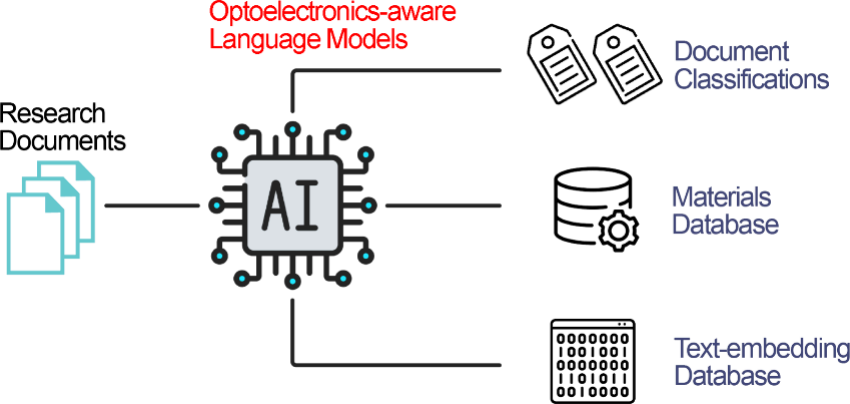

Pretrained language models have demonstrated strong capability and versatility in natural language processing (NLP) tasks, and they have important applications in optoelectronics research, such as data mining and topic modeling. Many language models have also been developed for other scientific domains, among which Bidirectional Encoder Representations from Transformers (BERT) is one of the most widely used architectures. We present three “optoelectronics-aware” BERT models, OE-BERT, OE-ALBERT, and OE-RoBERTa, that outperform both their counterpart general English models and larger models in a variety of NLP tasks about optoelectronics. Our work also demonstrates the efficacy of a cost-effective domain-adaptive pretraining (DAPT) method with RoBERTa, which significantly reduces computational resource requirements by more than 80% for its pretraining while maintaining or enhancing its performance. All models and data sets are available to the optoelectronics-research community.

## Introduction

Optoelectronics is a vibrant and fast-evolving field of research, where new scientific and technological breakthroughs are being published at an unprecedented pace. Its subfields, such as photovoltaics and light-emitting diodes, have attracted particular attention given their impact in energy-sustainable technologies. The vast volume of research literature coupled with the technical complexity of this subject matter is becoming increasingly prohibitive for scientists trying to retrieve knowledge and data of their interest efficiently so that they can stay up-to-date with the latest developments. Nonetheless, the research literature embeds invaluable knowledge which could drive innovations and accelerate scientific discoveries. The massive amount of information demands new approaches to fully exploit its potential.

**Figure 1:**
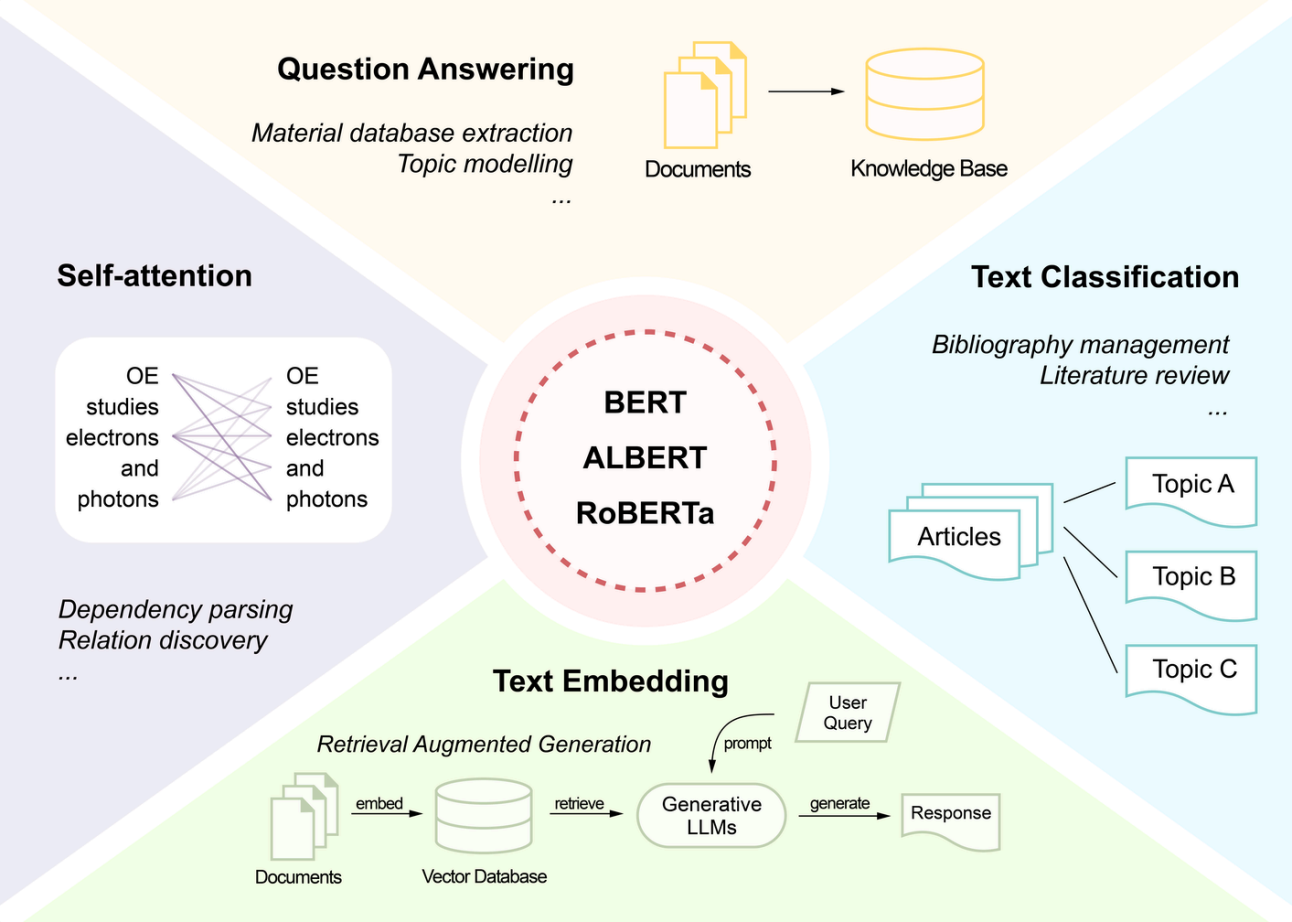
Applications of BERT-like models in academic research. **Left.** BERT-like models are built with the transformer architecture, which computes the attention between text tokens. The attention scores can be exploited to conduct dependency parsing and relation discovery. The models can also be fine-tuned to tackle specialized downstream tasks. **Top.** The question-answering (QA) capability of BERT-like models can be applied to tasks such as text-mining for material databases and topic modeling for research trend analysis. **Right.** The models can also be fine-tuned to perform text classification, which has wide application in literature review and bibliography management. **Bottom.** BERT-like models can be modified to produce contextual text embeddings that can be used in document retrieval based on the embedding similarities. The advancement in LLMs and RAG systems have also promoted the application of embedding models.

Recently, the rapid advancements in natural language processing (NLP) and language modeling (LM) techniques have been revolutionizing the way that scientific literature is interpreted and utilized. Such techniques have been successfully applied in various material-science domains, including database extraction for materials,^1−10^ enabling material discovery,^11^ and topic modeling.^12,13^ In these applications, both conventional NLP models and language models based on Bidirectional Encoder Representation from Transformer (BERT)^14,15^ have been investigated. In particular, transformer-based pretrained language models have been demonstrated to be more powerful and agile in various tasks, such as chemical named entity recognition (CNER)^16^ and abstract classification.^17^Figure 1 depicts the potential applications of BERT-like models in scientific research. With the stunning rise of generative large language models (LLMs),^18−20^ embedding technologies like BERT have attracted major interest for their capability in generating contextual text embeddings. Such embeddings allow powerful LLMs to acquire new unseen knowledge, without them needing to undergo expensive additional pretraining. This is achieved via retrieval augmented generations (RAGs) with an external vector database, where the new knowledge is encoded into vectors with these embedding models.^21−23^ Works have shown that domain specific embedding models afford better retrieval recall and precision which in turn lead to better text generation in a RAG system.^22,23^

Nonetheless, language models that have been trained on general English text corpora fail to perform equally well when they are applied to text about scientific work, where there are unseen highly specialized vocabulary, terminologies, academic writing conventions, and complicated sentence structures. Domain-specific language models have shown superior performance to their general English counterparts on domain-specific downstream NLP tasks.^4,17,24,25^ However, the computational cost to pretrain a domain-specific language model is prohibitive in many situations. Moreover, the amount of existing textual data in a given domain is usually much smaller than that which is required to pretrain a transformer-based language model. In the case of optoelectronics, we managed to gather a 5.7 GB plain text data set from published literature, which is much smaller than that which is used to create BERT (16 GB) and is even less than 5% of that which is associated with RoBERTa (160 GB).^14,26^ Hence, it is necessary to investigate alternative approaches of training domain-specific language models that are less computationally intensive and less data-hungry than that required for their full pretraining. The findings of such an investigation would also be potentially meaningful for practical and cost-effective domain adaptation into other areas of research in the natural sciences.

Thereby, language models that are “optoelectronics-aware” would make an important addition to the growing arsenal of scientific discovery workflows for optoelectronics. As shown in Figure 1, language models can be used in various stages of academic research, such as material data-mining, journal article classification and summarization, research trend analysis, factual question answering, and incorporation with LLMs in RAG systems. Meanwhile, it is also critical to reduce the training and running cost of these language models so that they are accessible to researchers with limited computation resources. Accordingly, this study explored the use of domain adaptation methods in the field of optoelectronics via their application to a family of BERT-like models, namely, BERT, RoBERTa, and ALBERT.^14,26,27^ Thereby, their “optoelectronics-aware” variants, OE-BERT, OE-RoBERTa, and OE-ALBERT were produced. Their performance characteristics on downstream tasks of abstract classification, extractive question-answering (QA), and text embedding and retrieval tasks were evaluated, where comparisons were also made against available language models that have been created for general English tasks as well as other material-science adapted language models.

## Methods

**Figure 2:**
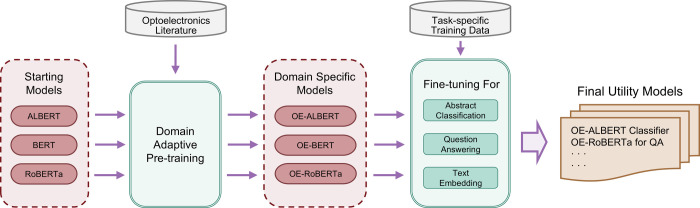
Training pipeline for the optoelectronics-adapted language models developed in this study. The starting BERT-like models, pretrained on general English text (ALBERT, BERT, and RoBERTa) were domain adaptive pretrained on optoelectronics research literature, yielding the OE-adapted models. Each resulting OE model was then fine-tuned on three downstream tasks: abstract classification, question-answering, and text-embedding, using task-specific training data sets. The resulting nine models represent the final utility models; such as the OE-RoBERTa model that serves for QA or the OE-ALBERT model that serves for abstract classification.

The full training pipeline for the optoelectronics-adapted BERT-like models is illustrated in Figure 2. The three BERT-like models, pretrained on general English text, were domain-adapted with optoelectronics research literature to produce OE-ALBERT, OE-BERT, and OE-RoBERTa, respectively. Each adapted language model was then fine-tuned on three downstream tasks: abstract classification, question-answering, and text embedding, respectively, which resulted in the final utility models. The detailed methods adopted in each stage of this training pipeline are introduced in the following subsections.

### Domain Adaptive Pretraining (DAPT)

#### Training Corpora

Optoelectronic literature was retrieved from Elsevier and the Royal Society of Chemistry (RSC). The search query was constructed with the Boolean logic (“optoelectronics” OR “light-emitting diode” OR “photovoltaics”) AND (“wavelength”) using appropriate wildcards, plurals, and case-sensitivity considerations, and applied to the search engine of each publisher. “light-emitting diode” and “photovoltaics” keywords were explicitly included in the query phrase, as they are sufficiently large and well-established subfields that many research papers in these domains do not mention “optoelectronics” in their text. For instance, searching only for the phrase “optoelectronics” gave 25.7k results from the RSC search engine, whereas a search query including all three phrases gave 43.6k results. The requirement of “wavelength” being in the search query was designed to exclude papers that mention “optoelectronics” where the scientific content are only loosely relevant. A total number of 192k publications were retrieved from both publishers. The retrieved contents were converted into plain text and cleaned using ChemDataExtractor,^1,2^ resulting a 5.7 GB plain text training corpus. The corpus is roughly one-third in size of the data set that was used to pretrain BERT and ALBERT (16 GB), whereas, the training corpus used to create the original RoBERTa is as large as 160 GB.

#### Model Architectures

Our three “optoelectronics-aware” language models, OE-BERT, OE-ALBERT, and OE-RoBERTa, were created starting from the base-size models of BERT, ALBERT, and RoBERTa, respectively,^14,26,27^ as shown in Figure 2. The three models all contain 110 M parameters in total, whose structures are almost identical to those described by Devlin et al.,^14^ while the minor differences in their tokenizers are summarized in Table 1. BERT and ALBERT use the same WordPiece tokenizer while RoBERTa has a Byte-Pair Encoding (BPE) tokenizer, which is more similar to that used by the generative pretrained transformer model GPT-2. Besides, ALBERT has adopted a factorized embedding parametrization strategy and a cross-layer parameter sharing strategy that reduces the total number of free parameters and memory consumption during computation.^27^ However, the training and inference speed of base-size ALBERT is about the same as BERT and ALBERT, since the total numbers of parameters of the three models are similar. In addition, the OpticalBERT-cased model was used as one of the reference language models, which has been domain adapted using literature about optical materials starting from the base-size BERT-cased model.^17^

**Table 1:** Summary of Differences in Case-Sensitivity, Tokenizers, and Vocabularies of the Three “Optoelectronics-Aware” BERT-like Models Studied

Model	Case Sensitive	Tokenizer	Vocabulary Size
OE-BERT	Uncased	WordPiece	30,000
OE-ALBERT	Uncased	WordPiece	30,000
OE-RoBERTa	Cased	Byte-Pair Encoding	50,000

#### Domain Adaptive Pretraining (DAPT)

The HuggingFace Transformers^28^ implementations of the three starting language models models: BERT, ALBERT, and RoBERTa, were used while DAPT was carried out using the Polaris supercomputing cluster at the Argonne Leadership Computing Facility (ALCF), Illinois, USA. These language models underwent DAPT through the Masked Language Modeling (MLM) task with dynamic masking^26^ and multinode distributed training was implemented and deployed with DeepSpeed, achieving an overall batch size of 4096. All three models were domain-adaptive pretrained for 10 epochs on the optoelectronics training corpus, corresponding to approximately 6,000 steps as suggested.^25^ Other pretraining hyperparameters can be found in the Supporting Information.

### Fine-Tuning for Downstream Tasks

#### Fine-Tuning for Abstract Classification

**Figure 3:**
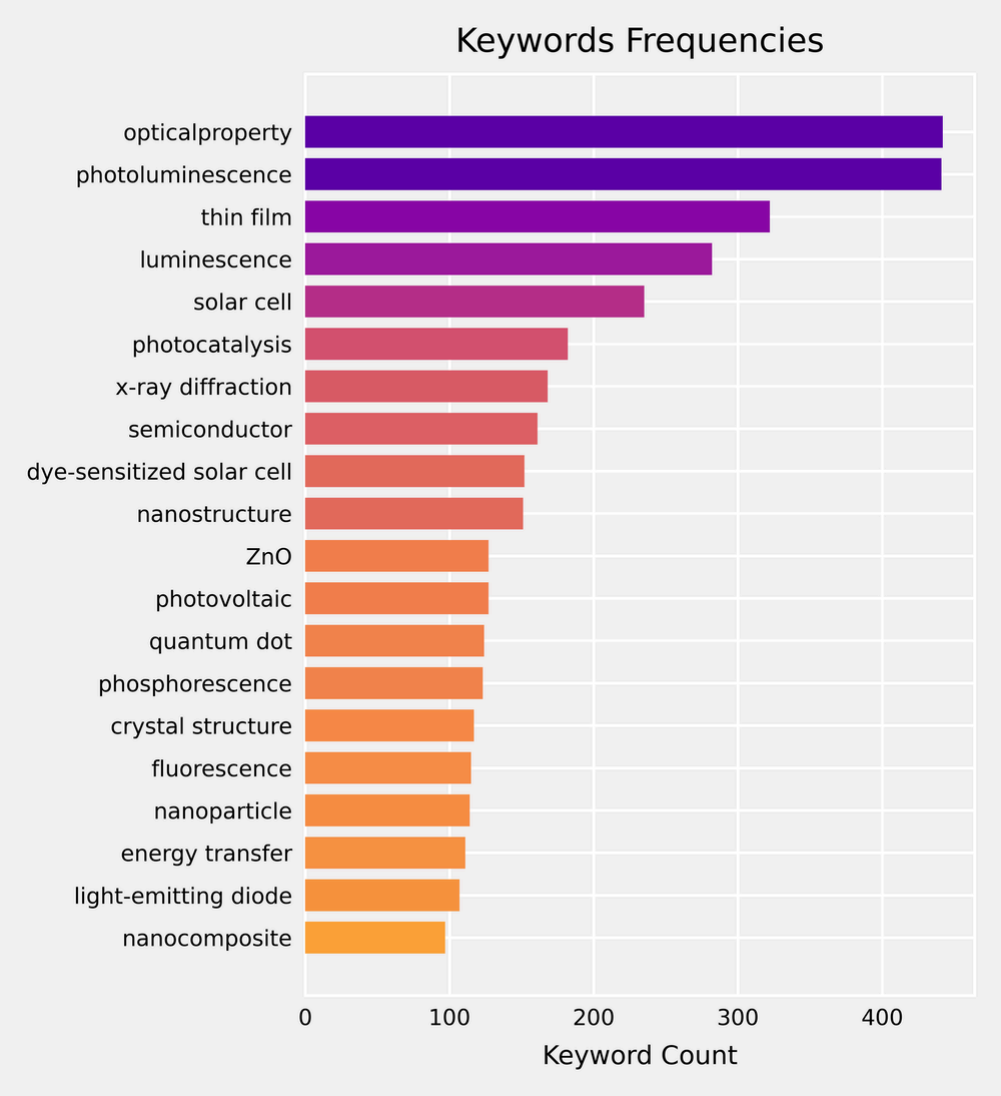
Keyword frequency bar chart for normalized keywords found in 20k Science Direct publications about optoelectronics These 20 normalized keywords outlined three major mutually exclusive categories in terms of material functions: light-emitting, light-harvesting, and photocatalysis categories.

A classification data set EHC-10k was created with three partitions of three distinct material functions, that concern, light-emitting (E), light-harvesting (H), or photocatalysis (C) categories. This choice of classes was a result of a keyword frequency analysis on 20k randomly selected publications about optoelectronics research. The keywords associated with the publications were retrieved using the Scopus Search API. Subsequently, these keywords were normalized and grouped according the length of the longest common substring, and occurrence frequencies of the keywords were calculated. The frequency plot of the top-occurring keywords is given in Figure 3. The three selected classes corresponded to the three largest subdomains with small intersection within the topic of optoelectronics according to the analysis. Following the above observations, the EHC-10k classification data set was collected from Scopus with 3,200 (abstract, title) pairs in each class, where an example was sampled into a class if it contained a keyword that fell into that class.

The EHC-10k data set was split in a 7:1 ratio into a train and validation set, respectively. The language models being studied were fine-tuned on the train set and evaluated on the out-of-sample validation set.

Meanwhile, a case study was designed to imitate the real-world application of the classification models by testing them on the top cited research articles published in 2023 that contain “optoelectronics” in their titles, abstracts, or keywords. In total, an imbalanced test set of 72 publications was gathered. There were 18 articles that did not fall in to the three named classes (EHC), while the other 54 could be classified into exactly one of the three classes.

The classification model structure followed the implementation described by Devlin et al.,^14^ where a dense multiclass classification layer was added on top of the BERT model. The input sequences were truncated when they exceeded the 512-token context window size of the language models. The output embedding of the [CLS] token was passed into the classification layer and the model was optimized to maximize the output probability of the correct class label.

#### Fine-Tuning for Question-Answering (QA)

The “optoelectronics-aware” language models were fine-tuned on the SQUAD v1.1 data set^29^ for extractive question-answering ability. This data set contains 100k crowd-sourced examples of question-answering with a general English language context.

We followed the HuggingFace Transformers implementation in approaching the QA task. Specifically, a linear layer was added over each pretrained OE language model such that it took the context and question of a training example as the input and provided an output of two 1-D vectors comprising start logits and end logits, respectively. The fine-tuning objective was set to minimize the cross-entropy loss between the predicted and ground-truth start and end positions of the answer.

The ability of each model to extract optoelectronic information was tested on a QA data set, TADF-numeric, which was sourced from a large material database that we had created previously using the chemistry aware NLP toolkit, ChemDataExtractor.^1,2^ This data set contains 231 examples of numerical questions spanning four key properties of thermally activated delayed fluorescence (TADF),^7^ which are emission wavelength, photoluminescence quantum yield, singlet–triplet energy split, and delayed fluorescence lifetime. In comparison to the test cases employed by Zhao et al. on another BERT model trained for the optical materials domoain,^17^ most of our examples contain names of organic molecular materials that are more diverse and versatile. Our test cases included properties with various dimensions and units that span several orders of magnitude, which makes the task more difficult to complete than the dimensionless refractive indices and dielectric constants that were of concern to Zhao et al.^17^

#### Fine-Tuning for Text Embedding and Retrieval

The original BERT language model was also pretrained on the next sentence prediction (NSP) task besides the masked language modeling (MLM) task, where it was trained to predict whether the two input sentences are consecutive or not.^14^ This capability can be readily used to compute text similarity for retrieval. Nonetheless, the computational cost for applying this method to retrieve texts from a large collection of text passages is prohibitive. So, we adopted the Sentence-BERT structure introduced by Reimers and Gurevych to alleviate this obstacle.^30^

BERT-like models naturally encode input texts into embedding vectors as their last hidden states, but the raw embeddings resulting from the MLM pretraining were not readily suitable for text retrieval. Hence, additional neural-network layers were added to fine-tune our “optoelectronics-aware” language models for better text retrieval embeddings. First, the raw embeddings generated by the pretrained model were transformed via a linear dense layer into the same dimension and activated with a tanh function. Second, the output embedding was computed by mean pooling the activated results. Finally, the textual similarity between two paragraphs *q* and *d* was computed using the cosine similarity distance, given by
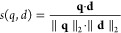
1where the text similarity increases as the metric moves from 0 to 1.

Fine-tuning language models for text similarity requires training data that are prepared in such a way that strongly relevant texts are tagged with the same label in contrast to other nonrelevant texts. Data sets of title-abstract pairs of journal articles have been widely used as high quality labeled ground-truths to fine-tune embedding models.^31,32^ In order to train an efficient embedding model for the optoelectronics literature, a data set OE-Ttl-Abs-303k of 303k pairs of journal titles and abstracts about optoelectronics was collected via the Scopus API, and then used to fine-tune the language models for text retrieval based on cosine similarity. There are about 0.6% of the abstracts from OE-Ttl-Abs-303k overshoot the 512-token context windows of our optoelectronics-aware language models. As the proportion of long abstracts is vanishingly low, they are truncated down to 512 tokens when used in fine-tuning and evaluations.

A contrastive learning strategy was implemented for fine-tuning the language models for text similarity in this study. Specifically, we used the InfoNCE loss^33^ as the model optimization objective, which is given as
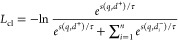
2where *s*(*q*, *d*) estimates the similarity between two pieces of text *q* and *d* as defined in eq 1. d^+^ is the positive example related to *q*, which together form a corresponding *d*-*q* abstract-title pair in the collected data set, while *d*^–^ is a negative example regarding *q*.

In this work, since the training data set contains as many as 303k examples, we adopted the in-batch random negative sampling approach to collect negative examples as described forthwith. For a title *q*_*j*_ within a batch of abstract-title pairs, all other abstracts *d*_*i*≠*j*_ are used as negative examples with respect to *q*_*j*_. Furthermore, the data-loading process has been designed to regenerate the random batches every epoch, which ensured that a large number of random negative abstracts were assigned to each title. In this study, four distributed processes were used each having a batch size of 128. The losses and gradients were computed individually in each process and then averaged for a back-propagation step.

The data set was split into a 9:1 train:test ratio. During evaluation, a given language model was required to retrieve the correct abstract (*d*) that corresponds to a title (*q*) according to the their embedding vectors. We quantified the performance using the recall@k metric for a single test query *q*_*i*_, defined as
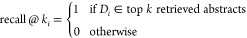
3with the overall being scoring computed as
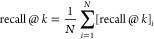
where *N* is the number of samples in the test set. The overall metric can be intuitively interpreted as the probability of retrieving the correct document within the top *k* choices.

## Results

### Pretraining

**Figure 4:**
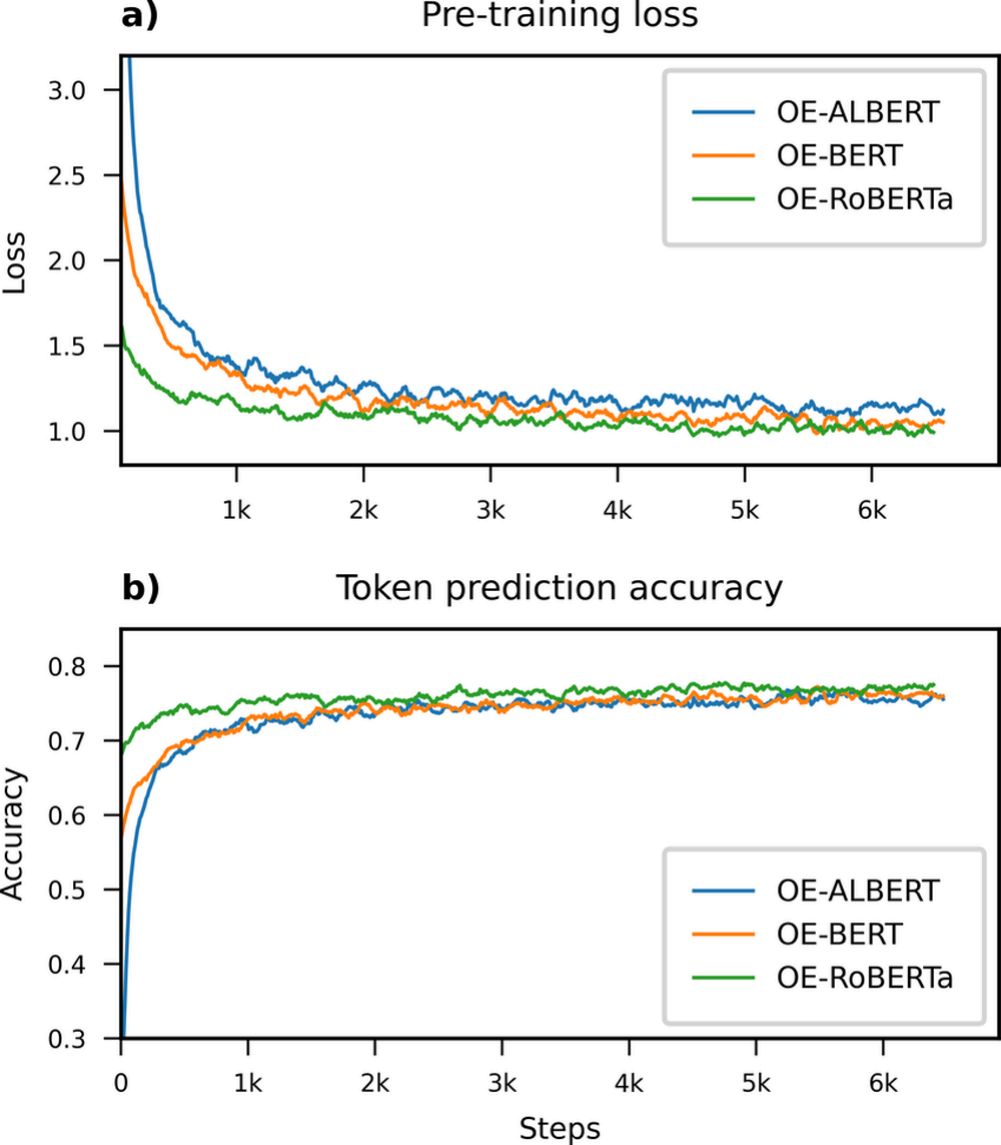
Pretraining loss and token prediction accuracy progress of BERT, ALBERT, and RoBERTa when further pretrained on optoelectronics literature in order to produce OE-BERT, OE-ALBERT and OE-RoBERTa, respectively. All three language models achieved a similar masked token prediction accuracy at the end of the DAPT process, while their progress of loss values are more separated with the OE-RoBERTa model affording the lowest loss value.

The domain-adaptive pretraining for each of the three optoelectronics-aware language models took roughly 3 h on 64 NVIDIA A100 40GB GPUs. In comparison, it took 8 days to pretrain OpticalBERT on eight A100 GPUs,^17^ which is equivalent to eight times more computational time than language models in this work. The loss and token prediction accuracy progress during the DAPT of the BERT, ALBERT, and RoBERTa models are plotted in Figure 4. At the end of DAPT process, the three “optoelectronics-aware” language models achieved similar performance in the masked language modeling pretraining task in terms of token prediction accuracy (78%). In contrast, their loss curves are separated more clearly throughout DAPT, with RoBERTa achieving the lowest loss values. Moreover, RoBERTa demonstrated much faster convergence than BERT and ALBERT. These observations could be attributed to that RoBERTa had been pretrained deeper on a much larger text corpus, giving it a better starting state than the other two language models.

### Case Study: Probing the Masked Language Modeling

How well do the language models understand optoelectronics? Since the language models are pretrained to predict masked tokens in text, some intuitive comparisons can be made between models that have been trained on optoelectronics texts and those that have not. We chose OE-RoBERTa and the original RoBERTa model for such as comparison and setup the two models to fill masked tokens of numerical values, units, and specialized terminologies in sentences, with two examples from each type of token. The detailed results are listed in Table 2. As can be seen from the top half of Table 2, OE-RoBERTa was able to fill in plausible tokens for all six masked tokens, which is a clear evidence of the language model acquiring domain-specific knowledge through the DAPT process. In contrast, the original RoBERTa model only managed to fill the unit tokens correctly and failed to fill the tokens of values and scientific terminologies. This can be explained by the fact that unit phrases are relatively more common in general English than the other two test cases.

**Table 2:** Relative Performance of the Optoelectronics-Aware OE-RoBERTa versus RoBERTa Language Models on Predicting Masked Tokens That Require Domain Knowledge from Optoelectronics^a^

Model	Sentences	Tokens predicted
OE-RoBERTa (optoelectronics-aware)	Red light has wavelength of _________ nm.	660, 630, 620, ...
	Carbon has an atomic number of _________.	6, 4, 10, ...
	The band gap has a size of 1 _________ volt.	electron, e, k, ...
	The photoluminescence quantum yield is above 90 _________.	%, %, percent, ...
	DFT is density _________theory.	functional, function, functional, ...
	_________ beam epitaxy is abbreviated as MBE.	Molecular, molecular, cular, ...
RoBERTa	Red light has wavelength of _________ nm.	100, 10, 300, ...
	Carbon has an atomic number of _________.	10, atoms, 5, ...
	The band gap has a size of 1 _________ volt.	electron, meter, 1/2, ...
	The photoluminescence quantum yield is above 90 _________.	%, percent, nm, ...
	DFT is density _________ theory.	ratio, reduction, gradient, ...
	_________ beam epitaxy is abbreviated as MBE.	Brain, Metal, Blue, ...

a Our “optoelectronics-aware” OE-RoBERTa model was able to fill all six spaces with reasonable tokens. In contrast, the RoBERTa model, which has not been domain adapted with optoelectronics knowledge, only managed to fill unit tokens and failed in filling numerical and terminological tokens.

### Abstract Classification

Abstract classification for scientific publication is an elemental NLP task which has wide application in literature review and bibliography management, helping researchers to quickly gather research works of interest. The aforementioned keyword analysis (cf. Figure 3) showed that there were three important and mutually exclusive subfields in optoelectronics, namely light-emitting (E), light-harvesting (H), and photocatalysis (C). We therefore fine-tuned the OE-adapted models and the original general English language pretrained counterpart models to classify optoelectronic publications into the three named categories. All models were fine-tuned and evaluated on the EHC-10k data set, the results of which are summarized in Table 3, where the standard errors are evaluated by fine-tuning the models from three random initializations. The OE-adapted models demonstrate better accuracy than their respective unadapted models by at least one standard error. The worst performing adapted model, OE-ALBERT, still achieved comparable accuracy to the best unadapted model. Among the OE-adapted models, OE-RoBERTa outperformed the other two with statistical significance, but the absolute improvements are small, being 0.3% and 0.5% relative to OE-BERT and OE-ALBERT, respectively.

**Table 3:** Classification Precision Scores of the Language Models When Tested on the Out-of-Sample Validation Set from the EHC-10k Dataset^a^

Classification Model	Validation Precision
OE-BERT	0.9770(2)
OE-ALBERT	0.9753(5)
OE-RoBERTa	**0.9797(2)**
BERT	0.975(1)
ALBERT	0.9686(2)
RoBERTa	0.976(1)

a The top section presents the results from the OE-adapted language model classifiers, while the bottom section shows the results from their general-English language pre-trained counterpart models.

### Case Study: Classifying Titles and Abstracts of the Top Cited Optoelectronics Papers in 2023

The aforementioned classification models were used to classify the top cited optoelectronics publications in 2023 in order to explore the extent by which the language models can be applied to real-world problems. In addition to the categories of light-emitting, light-harvesting, and photocatalysis, an undefined class of “other topics” was added to accommodate titles and abstracts of other research topics. Since the classification models were fine-tuned only on the three defined classes, modifications were needed to address the additional class of “other topics”. The final layer of each classification language model affords three logits in its output, which indicate the relative likelihood of a given input (title, abstract) being associated with an E, H, or C class. Therefore, a threshold value can be chosen for each classification language model such that only those (title, abstract) pairs with a logit larger than the threshold are categorized into one of the three defined classes. In such way, the language models can put titles and abstracts with low likelihood into the class of “other topics”. Furthermore, as in many real world situations, the test examples are imbalanced among the four classes, including the “other topics” class and the precision metric will be biased due to unequal distributions of examples. Hence, the weighted *F*_1_ metric was adopted in the evaluation of this test case. The metric is defined as,
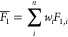
4where *w*_*i*_ is the relative weight of the *i*^th^ class and the expression is summed over all four classes.

The evaluation results are summarized in Table 4. By comparing between the two columns, the classification results were consistently better when the language models were fed with both abstracts and titles than abstracts alone. This observation indicates that additional information is contained within titles that complement the abstracts. Within each 3 × 1 column subsection of Table 4, the performance of the BERT-, ALBERT-, and RoBERTa-based classification language models was similar. Nonetheless, it was surprising that RoBERTa-based models performed slightly poorer than the BERT- and ALBERT-based models by one to two documents, even though it achieved better precision on the validation set of EHC-10k. We hypothesized that this finding is a consequence of the fact that both BERT- and ALBERT-based models use uncased vocabularies while RoBERTa-based models employ a cased vocabulary; and that the upper-case words in this test set contained misleading information to the language models. We checked this hypothesis by fine-tuning the OE-RoBERTa model with a case-lowered EHC-10k data set; its performance on this case study is given in the final row in Table 4. The OE-RoBERTa classification model produced with fine-tuning on the case-lowered EHC-10k data set shows improved  scores relative to the results from the cased RoBERTa or OE-RoBERTa models and it was also the best achieving model when tested against both abstract and abstract+title scenarios, which supports our hypothesis.

**Table 4:** Score Results of Classifying the Top Cited Optoelectronics Papers in 2023, Using the Classification Models Which Were Fine-Tuned from the “Optoelectronics-Aware” BERT-like Models and Their General English Counterpart Models

Domain adaptation	Classification Model	abstract (no. correct)	abstract + title (no. correct)
Adapted	OE-BERT	0.891 (64)	0.915 (66)
	OE-ALBERT	0.874 (63)	0.916 (66)
	OE-RoBERTa	0.876 (63)	0.902 (65)
Unadapted	BERT	0.862 (62)	0.889 (64)
	ALBERT	0.846 (61)	0.875 (63)
	RoBERTa	0.856 (61)	0.866 (62)
Case-lowered	OE-RoBERTa	0.918 (66)	0.918 (66)

**Figure 5:**
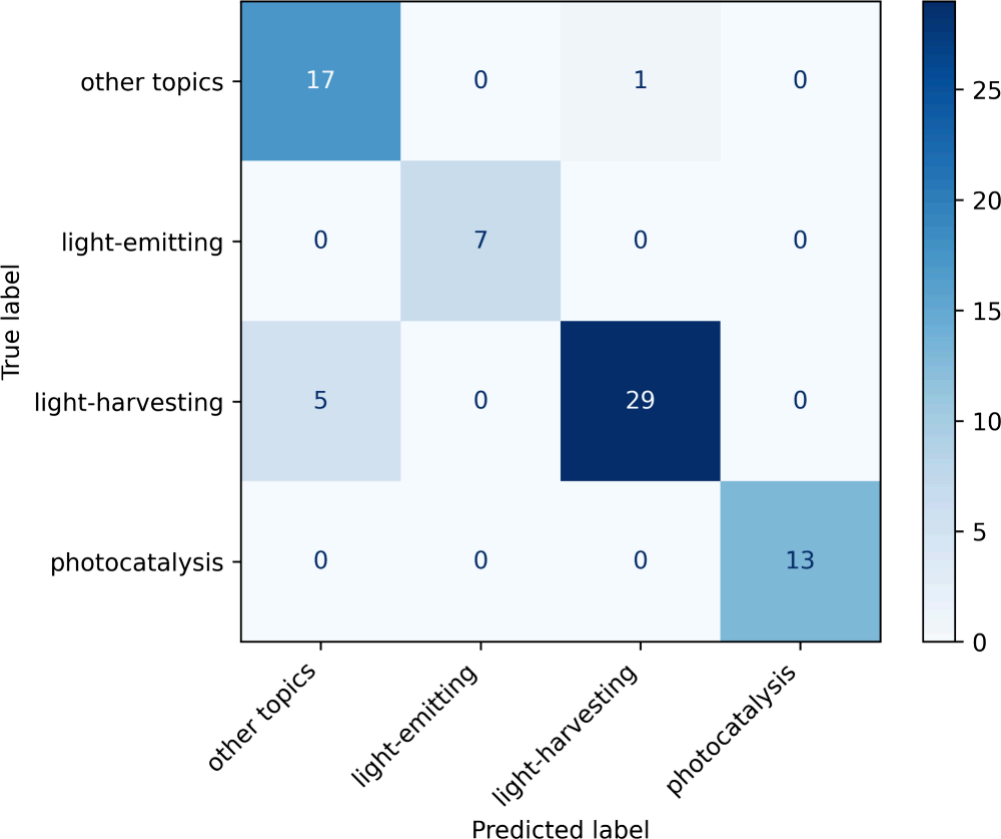
Confusion matrix of the classification results on titles concatenated with abstracts from top cited optoelectronics papers in 2023, using the OE-RoBERTa case-lowered classification model. Most confusions occur between the “light-harvesting” class and the “other topics” class.

Figure 5 illustrates the confusion matrix for the result achieved by the OE-RoBERTa case-lowered classification model. It can be seen that all six confusions occur between the “other topics” class and the “light-harvesting” class. Three of the five mis-classified abstracts in the “light-harvesting” class had been classified correctly as “light-harvesting”, but the predicted logits were lower than the confidence threshold and hence they were categorized into “other topics” erroneously. The only one mis-classified example in the “other topics” class which was classified as “light-harvesting”, occurs because the corresponding paper investigates a family of thin-film materials and simply mentions their potential application to photodetectors and photovoltaics, which led to the confusion. In summary, the OE-RoBERTa case-lowered classification model showed little confusion among the three explicitly defined classes and it also classified 17 out of 18 abstracts that are of undefined “other topics”. This demonstrated the better extensibility of pretrained language models to unseen situations over conventional NLP techniques.

### Question Answering

#### Performance of Optoelectronics-Aware BERT-like Models on SQuAD v1.1

Six language models were fine-tuned on SQuAD v1.1 for the question-answering task using the HuggingFace implementations; the three “optoelectronics-aware” models: OE-RoBERTa, OE-BERT, and OE-ALBERT; and three reference models: RoBERTa-large, RoBERTa-base, OpticalBERT-cased.^17,26^ Their performance characteristics on the SQuAD v1.1 validation set are presented in Table 5. The Exact Match (EM) and *F*_1_ metrics were adopted to evaluate their performance in the validation set.^29^ The EM score of an QA example is 1 if the answer from the language model is identical to the ground-truth answer and 0 otherwise. The *F*_1_ score is a smoother measure of the answer’s quality, defined as
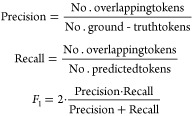
5where *F*_1_’s lowest possible value is 0, if either the precision or the recall is 0, and its highest possible value is 1.0, which means perfect precision and recall for all tokens in the ground-truth answer. The overall EM and *F*_1_ scores are the respective average scores across the validation set.

**Table 5:** Exact Match and *F*_1_ Score Results on the Test Set of SQuAD v1.1, Using the QA Models which Were Fine-Tuned from the “Optoelectronics-Aware” BERT-like Models and Their General English Counterpart Models^a^

QA Model	Exact Match	*F*_1_
RoBERTa-large	**0.882**	**0.943**
RoBERTa-base	0.853	0.920
OpticalBERT-cased	0.818	0.890
OE-RoBERTa	0.837	0.911
OE-BERT	0.794	0.871
OE-ALBERT	0.800	0.882

a The RoBERTa-large QA model afforded the highest scores in both metrics on this general English-language QA task.

RoBERTa-large performed much better than the other five base-size models in this validation set owing to its incorporation of a much larger number of parameters. By comparing the results of the unadapted RoBERTa-base and the adapted OE-RoBERTa QA models, it is evident that the domain adaptation into optoelectronics had a negative impact on the model’s capability in this task of general English QA, with −1.6% in EM score and −0.9% in *F*_1_ score. In contrast, the OE-RoBERTa QA model achieved around 2% higher exact-match and *F*_1_ scores than the OpticalBERT-cased QA model.^17^ A possible explanation of this finding is that the OpticalBERT-cased model had been domain adapted for 70k steps, normalizing to a batch size of 4096, using literature about the broader topic of optical materials, which is many more than the 6k steps that were used in the DAPT process that produced the OE-RoBERTa model. Thus, the aforementioned negative impact on general English QA tasks is stronger for the OpticalBERT-cased model.

#### Extracting TADF Properties

The ability of these six language models to perform optoelectronic information extraction was further tested using the TADF-numeric QA data set. In order to investigate their detailed behavior against this test, we adopted an additional “*Contain*” score that is 1 if the model’s answer contains the ground-truth answer and 0 otherwise. This metric was designed to distinguish between completely irrelevant answers and answers that are correct but are not concise.

The performance results of the six language models on the TADF-numerical QA data set are listed in Table 6; noting that trailing spaces, periods, commas, and brackets in answers were removed from the models’ answers before passing to the evaluation. We first consider their performance levels when tested against questions that comprise plain text (Table 6, left panel). The OE-RoBERTa QA model achieved the highest EM score with at least a 15% improvement over the original RoBERTa owing to its domain adaptation to optoelectronics. The OpticalBERT-cased QA model, which was domain adapted into the broader topic of “optical materials” instead of “optoelectronics”, achieved the second highest EM score which is still 10% higher than that of the general English RoBERTa-large QA model. These observations demonstrate the necessity of a DAPT process, where smaller language models can significantly outperform larger models in domain-specific tasks even when their adaptation is topic-wise broad (i.e., optical research covers much more beyond optoelectronics).^24^ In contrast, the OE-RoBERTa QA model achieved better results on this in-domain QA task, having been realized by a DAPT process involving 6k steps, which was just about 9% of the number of the DAPT steps of the OpticalBERT-cased model. As discussed in Section 3.3.1, the OE-RoBERTa QA model also maintained a better performance on general English language QA tasks from SQuAD v1.1. This suggests that the RoBERTa model may provide a more capable and cost-effective starting point for domain adaptation into optoelectronics and other scientific domains compared to the BERT model and ALBERT model.

**Table 6:** Exact Match, *F*_1_, and *Contain* Score Results of Six Language Models on QA Tasks from Tests on the TADF-Numerical QA Dataset^a^

	Plain text questions			Added symbolic hints		
QA Model	Exact Match	*F*_1_	*Contain*	Exact Match	*F*_1_	*Contain*
OE-RoBERTa	**0.801**	**0.869**	0.909	**0.823**	**0.874**	0.922
OpticalBERT-cased	0.762	0.865	**0.913**	0.758	0.867	0.896
RoBERTa-large	0.649	0.791	0.896	0.723	0.844	0.922
OE-BERT	0.593	0.757	**0.913**	0.606	0.773	**0.952**
OE-ALBERT	0.606	0.750	0.827	0.628	0.767	0.862
RoBERTa-base	0.498	0.695	**0.913**	0.602	0.759	0.944

a The left panel shows the results when the QA language models were tested using questions consisting of plain text; the right panel summarizes the results when they were tested against the same set of questions but with additional symbolic hints inserted into the questions.

In sharp contrast, the OE-BERT and OE-ALBERT QA models perform much worse compared to not only the OE-RoBERTa QA model but also to the general English-language pretrained RoBERTa-base and RoBERTa-large QA models, even though they have undergone the same DAPT process as the OE-RoBERTa model. In addition to the factors associated with the nature of training corpora and the number of free parameters that have already been discussed, the fact that RoBERTa models use a BPE tokenizer may be another relevant factor. The BPE tokenizer had a larger vocabulary than those used in other language models presented in this work and could split characters at byte-level. This potentially improves the representation of scientific texts where there is a diverse use of new terminologies and unicode characters.

All language models except for OE-ALBERT achieve similar *Contain* scores (within ±1%), in contrast to their vastly different EM scores. In particular, the OE-BERT QA model achieves the highest *Contain* score while obtaining the lowest EM score among the OE-adapted QA models. The observation is a direct result of answers from the OE-BERT QA model being correct but containing too many unnecessary tokens (i.e., false positives). Moreover, the smaller RoBERTa-base QA model achieves a slightly better *Contain* score than the RoBERTa-large QA model, whereas RoBERTa-large is much more precise than the RoBERTa-base QA model as can be seen from their 15% different EM scores. These observations imply that all six investigated models can locate the span of texts that contains the correct answer despite the differences in model size and pretraining levels; while the extent by which answers to numerical questions were concise is was significantly improved when using domain adaptation and a model of increased size.

#### Ablation Studies on QA Performance

The effects of domain adaptation were further investigated by performing an ablation evaluation whereby the original questions from the TADF-numeric QA data set were posed to each language model, but with common symbolic specifiers inserted for these four properties, which are λ_max_, Φ, Δ*E*_ST_, and τ_D_. The same set of evaluation metrics were computed for these questions, which are listed in the right-most panel of Table 6. Comparing the results between the left and right panels of Table 6, all six models benefit from the additional symbolic hints inserted into the questions in at least one of the metrics. The observed performance improvements for the general English-language pretrained models, RoBERTa-base and RoBERTa-large, were as large as around 10% in EM and 5% in *F*_1_ scores, indicating that the symbolic specifiers provide helpful additional information for locating a more precise and accurate answer for these models, which have not seen domain-specific texts. In contrast, the improvements were much smaller for the domain adapted OE-BERT, OE-ALBERT, OE-RoBERTa, and OpticalBERT-cased models. This observation can be attributed to the fact that these models have learnt the mapping relations between the textual names of the TADF properties and their symbolic representations to some extent during the DAPT process.

**Figure 6:**
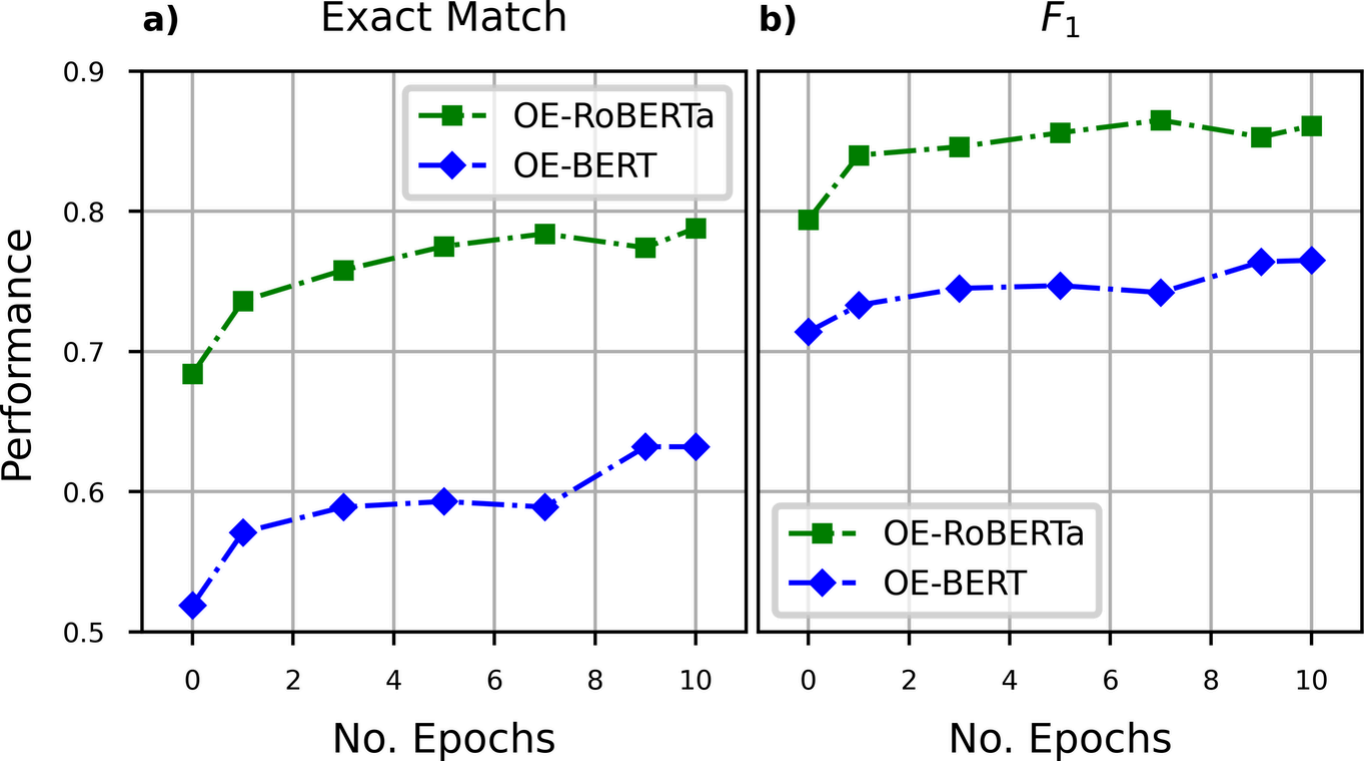
Performance evolution on the TADF-numerical QA data set of the OE-BERT and OE-RoBERTa models against the progress of their optoelectronics DAPT process as defined by the number of epochs of the DAPT process. **a)** The progressive evolution of the Exact Match metric. **b)** The progressive evolution of the *F*_1_ metric. The metric scores of OE-BERT and OE-RoBERTa evolves highly parallel for both Exact Match and *F*_1_.

An further ablation study was designed to explore the reasons for the vastly different performance between OE-BERT and OE-RoBERTa QA models on this TADF numerical QA task. Individual OE-BERT and OE-RoBERTa QA models were fine-tuned from seven checkpoints saved during their DAPT processes; their performance levels on the QA task were then evaluated and by plotting their EM and *F*_1_ scores against the progressive evolution of their DAPT process as defined by the number of epochs of the DAPT process. Figure 6 shows that the performance of the OE-RoBERTa QA model evolves in a fashion that is highly parallel to that of the OE-BERT model, and that the total level of improvements in the metrics due to the DAPT process was were also similar for both models. This observation supports our previous claim that the RoBERTa model is a more cost-effective model than the BERT model for domain adaptation under the same amount of additional training. The observed parallel behavior also indicates that the RoBERTa model does not learn faster than BERT; it simply has a better starting point. The similar rate of improvement can be explained by the fact that both models have the same hidden layer structures; hence, the speeds of model convergence are very similar.

### Text Embedding and Retrieval

Text retrieval forms a fundamental component of many NLP systems, such as search engines and retrieval augmented generation (RAG) pipelines. The performance of the retrieval stage also has a direct impact on downstream analysis in these systems (e.g., text generation with LLMs). As LLMs often suffer from the “lost in the middle” problem,^34^ it is of great importance to retrieve concise and relevant information for the LLMs during RAG. RAG systems have provided a new direction of how research literature can be used, and it could also bridge the gap between the highly specialized scientific knowledge and the capabilities of LLMs that are pretrained on more general English contents through text retrieval. Therefore, a document retrieval system specialized in optoelectronics text has high potential applied value when enabled with RAG and data-mining capabilities for optoelectronic research.

Thereby, the OE-adapted language models developed through this work can be fine-tuned into embedding models that can encode text about optoelectronics into vectors; these in turn serve for a text retrieval pipeline that retrieves relevant information given a query, according to the cosine similarities of these embedding vectors to the embedding vector of the query. Two embedding models were fine-tuned from the OE-RoBERTa model using 303k title-abstract pairs sourced from optoelectronics publications, which have been collected using the Scopus API. The transformer layers of one of these two embedding models were frozen and only its pooling layers were fine-tuned while all parameters of the other embedding model were fine-tuned. Two embedding models were also fine-tuned from the OE-BERT and OE-ALBERT models using the same data set, respectively. For comparison, the off-the shelf gte-base-en-v1.5 embedding model was also evaluated with the OE-Ttl-Abs-303k data set, without employing any training or alterations on the original model obtained from HuggingFace.^31^gte-base-en-v1.5 was also the top performing embedding model that had less than 250 M parameters in the MTEB (Massive Text Embedding Benchmark) Leaderboard at the time of access.^35,36^

**Figure 7:**
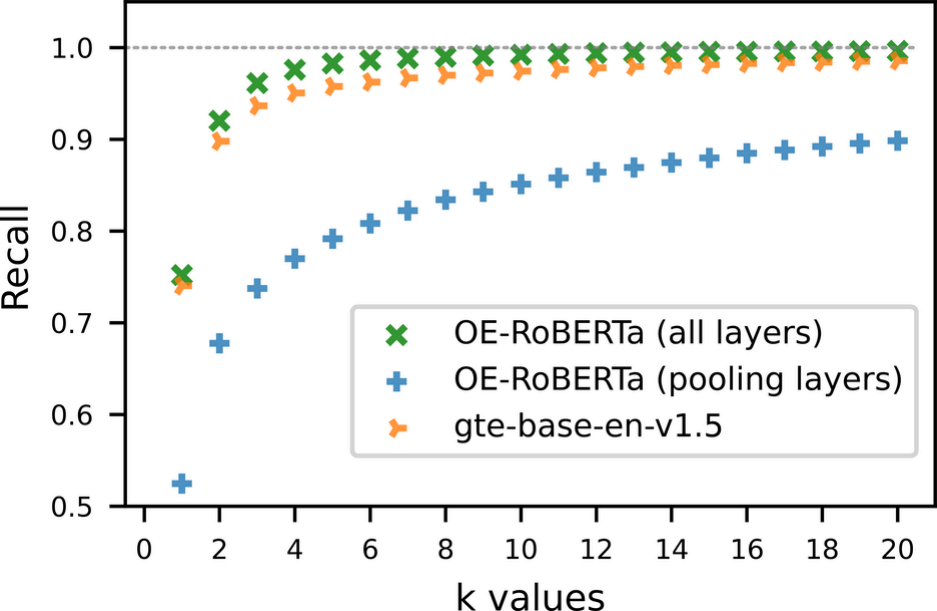
Recall*@k* results plotted against *k* for 1 ≤ *k* ≤ 20 when testing on the test set of the OE-Ttl-Abs-303k data set using the gte-base-en-v1.5 model, the OE-RoBERTa embedding model (all layers fine-tuned), and the OE-RoBERTa embedding model (pooling layers fine-tuned). The OE-RoBERTa embedding model that had all its layers fine-tuned outperformed the state-of-the-art embedding model that is of a similar size, gte-base-en-v1.5, on this optoelectronics in-domain retrieval task.

Table 7 presents the key values of the recall*@k* metric, defined in eq 3, resulting from all five investigated embedding models. In particular, the recall*@k*s of the gte-base-en-v1.5 model, the OE-RoBERTa embedding model with all layers fine-tuned, and the OE-RoBERTa embedding model with just its pooling layers fine-tuned are plotted against *k* in Figure 7. The recall*@k* metric measures the likelihood of retrieving the correct abstract within the top-*k* most similar abstracts, given a title. The OE-RoBERTa embedding model with all parameters fine-tuned outperformed the gte-base-en-v1.5 model on all *k* values, with recall values over 0.99 at *k* ≥ 9. In contrast, the OE-RoBERTa embedding model that had only its pooling layers fine-tuned afforded significantly lower recall values. The comparison indicates that the pretrained transformer layers need to be modified in order to obtain better text embeddings for text retrieval based on similarity. Moreover, the total cost of training the OE embedding model from the RoBERTa model is much lower than that required to train an embedding model from scratch. In comparison, the other two embedding models that were fine-tuned from the OE-BERT model and the OE-ALBERT model demonstrate lower recall values at all *k* values. The OE-ALBERT embedding model with all layers fine-tuned performed worse than the OE-RoBERTa embedding model with only the pooling layers fine-tuned. This can be attributed to the features of parameter-sharing and embedding size factorization of the ALBERT model architecture. Though these features significantly reduce the number of free parameters in ALBERT-based models while maintaining their overall sizes, they harm the performance when compared to other models, such as BERT-based and RoBERTa-based models, that are of similar overall size.

**Table 7:** Recall*@k* Values at *k* = 1, 5, 10, 20 Afforded by Each Invested Language Model When Tested on the Test Set of the OE-Ttl-Abs-303k Dataset

Embedding Model (fine-tuning level)	recall*@*1	recall*@*5	recall*@*10	recall*@*20
OE-RoBERTa (all layers)	0.752	0.983	0.992	0.996
OE-RoBERTa (pooling layer)	0.525	0.791	0.851	0.899
gte-base-en-v1.5 (reference model)	0.740	0.958	0.974	0.986
OE-BERT (all layers)	0.611	0.865	0.906	0.932
OE-ALBERT (all layers)	0.415	0.540	0.718	0.772

## Conclusions

This study has investigated a cost-effective method of domain-adaptive pretraining (DAPT) to produce language models that are tailored for optoelectronics applications. Three “optoelectronics-aware” language models, OE-BERT, OE-RoBERTa, and OE-ALBERT were realized by conducting DAPT on BERT, RoBERTa,and ALBERT models, respectively. Their performance on downstream tasks of abstract classification, question answering (QA), and text embedding and retrieval were evaluated using direct comparisons but also including performance comparisons against their counterpart models that were pretrained on general English-langauge as well as an OpticalBERT model.The “optoelectronics-aware” models demonstrated superior ability in dealing with unseen categories during abstract classification, where the best performing model correctly classified 17 out of 18 abstracts from unseen categories. In QA tasks that were assessed by information from thermally activated delayed fluorescence (TADF) properties, the OE-RoBERTa QA model achieved better results than the larger RoBERTa-large model and the OpticalBERT-cased model which had been pretrained for 12 times more steps. Nevertheless, OE-BERT and OE-ALBERT models performed much worse in comparison. The reason behind this appears to be that BERT and ALBERT models had been pretrained far less than RoBERTa. Regarding text embedding and retrieval, the vector embedding model fine-tuned from the OE-RoBERTa model beat the best general English-language embedding model of a similar size, gte-base-en-v1.5, on retrieving abstracts from optoelectronics papers given their titles. The improvements in the recall*@k* values are 1.5% and 2.5% at *k* = 1 and *k* = 5, respectively.

Our work has demonstrated that the use of the DAPT method can consume much lower computational resources while achieving similar or better performance on downstream tasks compared to models that received considerably more pretraining. We have also argued that the RoBERTa model offers a better starting-point model, rather than BERT or ALBERT, for domain adaptation into the field of optoelectronics as a result of its much larger pretraining corpus. The RoBERTa model may also benefit from its BPE tokenizer which has a larger vocabulary and byte-level splitting ability.

Another potential future application of these domain-specific language models could involve database pruning and enriching during text-mining of material data from scientific literature. Current material data-mining technologies still suffer from many difficulties such as missing laboratory conditions, merging data points of the same material, and not finding structural or compositional information, making the corresponding extracted database less systematic to use. Such a database could be enriched or pruned in a “retrieve and update” fashion, according to a vector database of research literature that could be embedded using the “optoelectronics-aware” language models. This would allow us to access information related to material data points at a more fine-grained level, which could potentially lead to more innovative applications of artificial intelligence in optoelectronics research.

## Data Availability

The pretrained language models and the fine-tuned language models for the abstract classification, question-answering task for text, and text-embedding for retrieval are available at https://huggingface.co/collections/CambridgeMolecularEngineering/ as are the corresponding fine-tuning and testing data sets for this study. Thereby, the abstract datasets are released as lists of their DOIs in order to respect copyright regulations.

## Supporting Information Available

List of acronyms and abbreviations used in the paper; pretraining and fine-tuning implementation details; and confusion matrices of the abstract classification case study (PDF)
