# Supplementary material for: Cost-Efficient Domain-Adaptive Pretraining of Language Models for Optoelectronics Applications
Source: J Chem Inf Model. 2025 Feb 11;65(5):2476–86. doi: 10.1021/acs.jcim.4c02029 (PMC11898057; doi:10.1021/acs.jcim.4c02029)
Supplement: Supplementary file 1 — ci4c02029_si_001.pdf [file ci4c02029_si_001.pdf]

Supporting Information: Cost-efficient domain-adaptive pre-training of language models for optoelectronics applications

Dingyun Huang[1] and Jacqueline M. Cole[1,2,*]

[1]ISIS Neutron and Muon Source, Rutherford Appleton Laboratory, Harwell Science and Innovation Campus, Didcot, Oxfordshire OX11 0QX, U.K.

[2]Cavendish Laboratory, Department of Physics, University of Cambridge, J. J. Thomson Avenue, Cambridge CB3 0HE, U.K.

[*]Author for correspondence: jmc61@cam.ac.uk (J. M. Cole)

January 20, 2025

Contents

1 Acronyms / Abbreviations **S2**

2 Implementation Details **S3**

2.1 Domain-adaptive pre-training S3

2.2 Fine-tuning S4

2.2.1 Fine-tuning for abstract classification S4

2.2.2 Fine-tuning for question answering S4

2.2.3 Fine-tuning for text embedding and retrieval S4

2.3 Confusion matrices of the classification results in the case study S5

1 Acronyms / Abbreviations

This section describes a list of abbreviations used in the manuscript. The order of these abbreviations in the list follows the order of those that appear in the manuscript.

- NLP: Natural language processing
- BERT: Bidirectional encoder representations from transformers
- DAPT: Domain-adaptive pre-training
- LM: Language modelling
- CNER: Chemical-named-entity recognition
- LLM: Large language model
- RAG: Retrieval-augmented generation
- QA: Question answering
- RSC: Royal Society of Chemistry
- BPE: Byte-pair encoding
- ALCF: Argonne Leadership Computing Facility
- EHC-10k: A classification dataset comprising 9,600 paris of abstracts and titles from research papers about optoelectronics, labelled into three categories according to material functions; these are light-emitting (E), light-harvesting (H), and photocatalysis (C).
- API: Application programming interface
- SQuAD: Stanford question answering dataset
- OE-Ttl-Abs-303k: A dataset comprising 303k pairs of abstracts and titles from research papers about optoelectronics.
- MLM: Masked language modelling
- TADF: Thermally-activated delayed fluorescence
- NSP: Next sentence prediction
- EM: Exact match
- MTEB: Massive text embedding benchmark

2 Implementation Details

2.1 Domain-adaptive pre-training

The training hyperparameters used in the DAPT process, which afforded our ”optoelectronics-aware” language models, are listed below:

- Number of layers: 12
- Hidden size: 768
- Number of self-attention heads: 12
- Maximum sequence length: 512
- Batch size: 4096
- Hidden layer dropout probability: 0.1
- Attention layer dropout probability: 0.1
- Optimizer: AdamW
- Adam β_1: 0.9
- Adam β_2: 0.98
- Adam ϵ: 1e-8
- L2 weight decay: 0.01
- Initial learning rate: 1e-5
- Warm-up ratio 0.06
- Learning rate schedule: Linear
- No. epochs: 10

2.2 Fine-tuning

2.2.1 Fine-tuning for abstract classification

Each OE-adapted model was fine-tuned on the EHC-10k dataset for three epochs with a batch size of 48. The initial learning rate was set to 8e-6 accompanied by a linear decay in the learning rate.

2.2.2 Fine-tuning for question answering

Each OE-adapted model was fine-tuned on SQuAD v1.1 for four epochs with a batch size of 64. The initial learning rate was set to 4e-5 accompanied by a linear decay in the learning rate.

2.2.3 Fine-tuning for text embedding and retrieval

Each OE-adapted model was fine-tuned on the OE-Ttl-Abs-303k dataset for ten epochs with a per-device batch size of 128 distributed over 4 devices. The initial learning rate was set to 2e-4 starting with a warm-up stage of 540 steps and followed by and a linear decay in the learning rate.

2.3 Confusion matrices of the classification results in the case study

All confusion matrices of the results from the classification case study were listed. The case study tested the ”optoelectronics-aware” classification models with the top-cited papers about optoelectronics in 2023, asking the models to categorize these papers into four classes: light-emitting, light-harvesting, photocatalysis, and other topics.

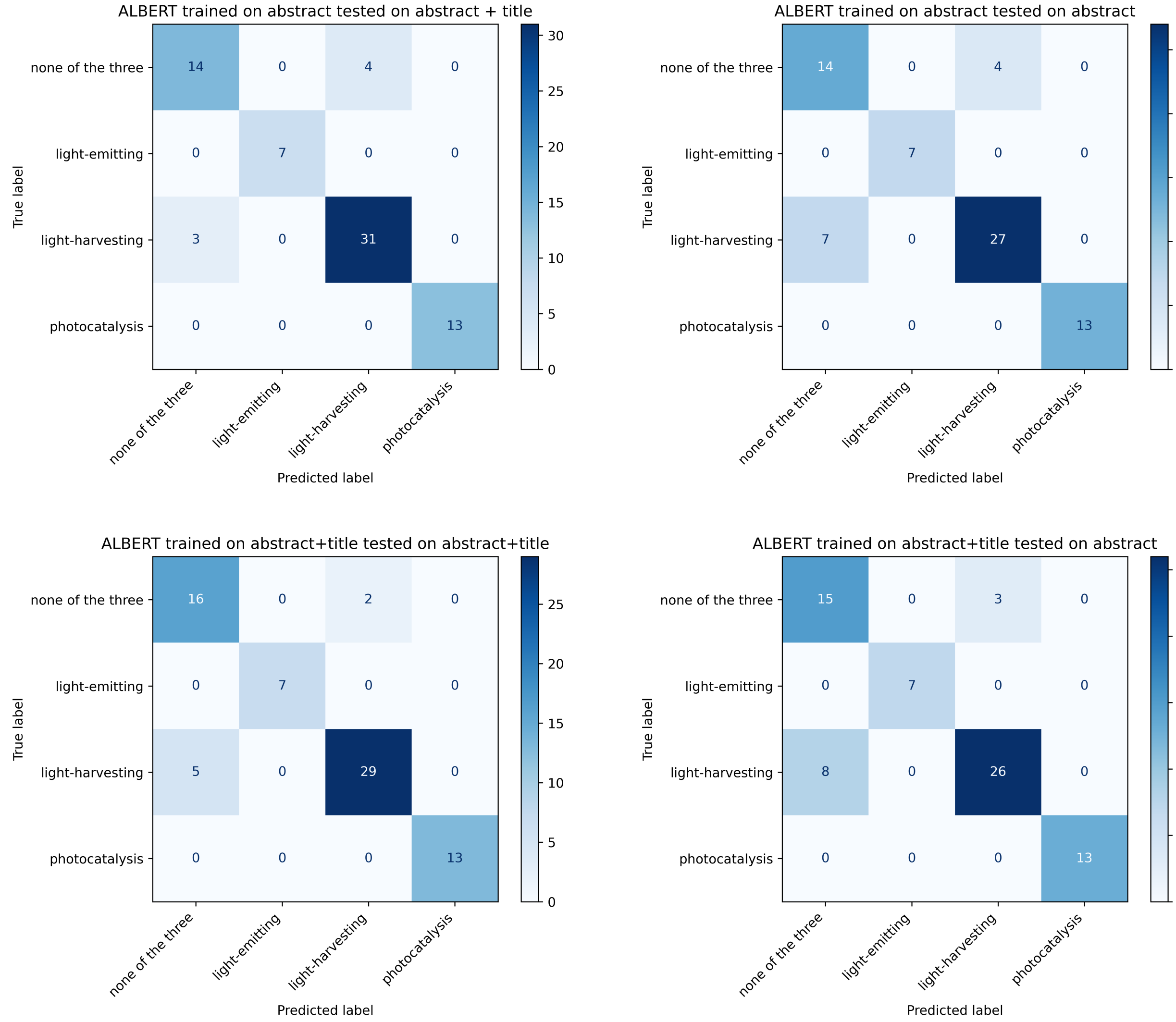

Figure S1: Confusion matrices of the results on the case study of classifying the 2023 top cited papers about optoelectronics, obtained with the OE-ALBERT classification model under four different setups.

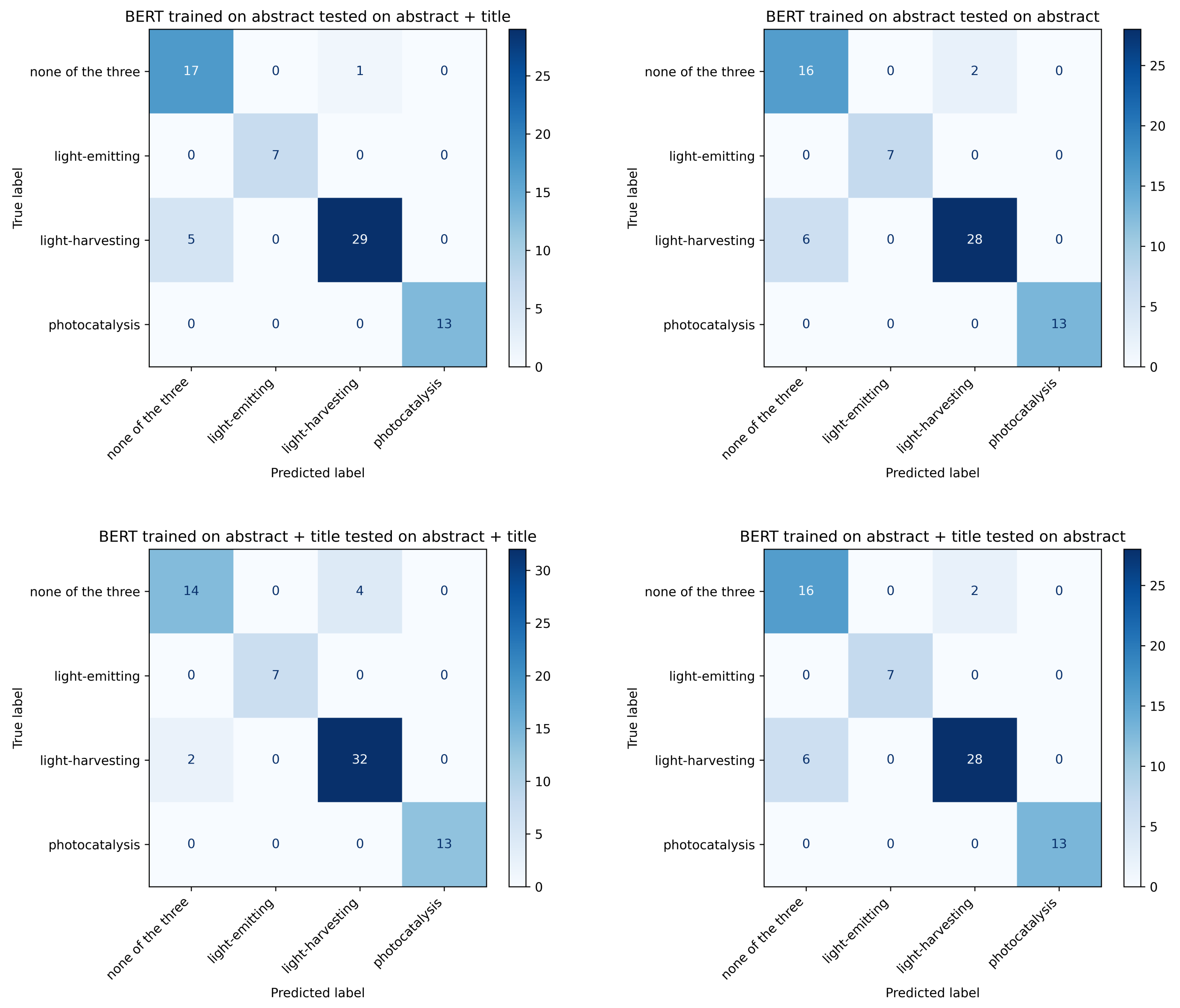

Figure S2: Confusion matrices of the results on the case study of classifying the 2023 top cited papers about optoelectronics, obtained with the OE-BERT classification model under four different setups.

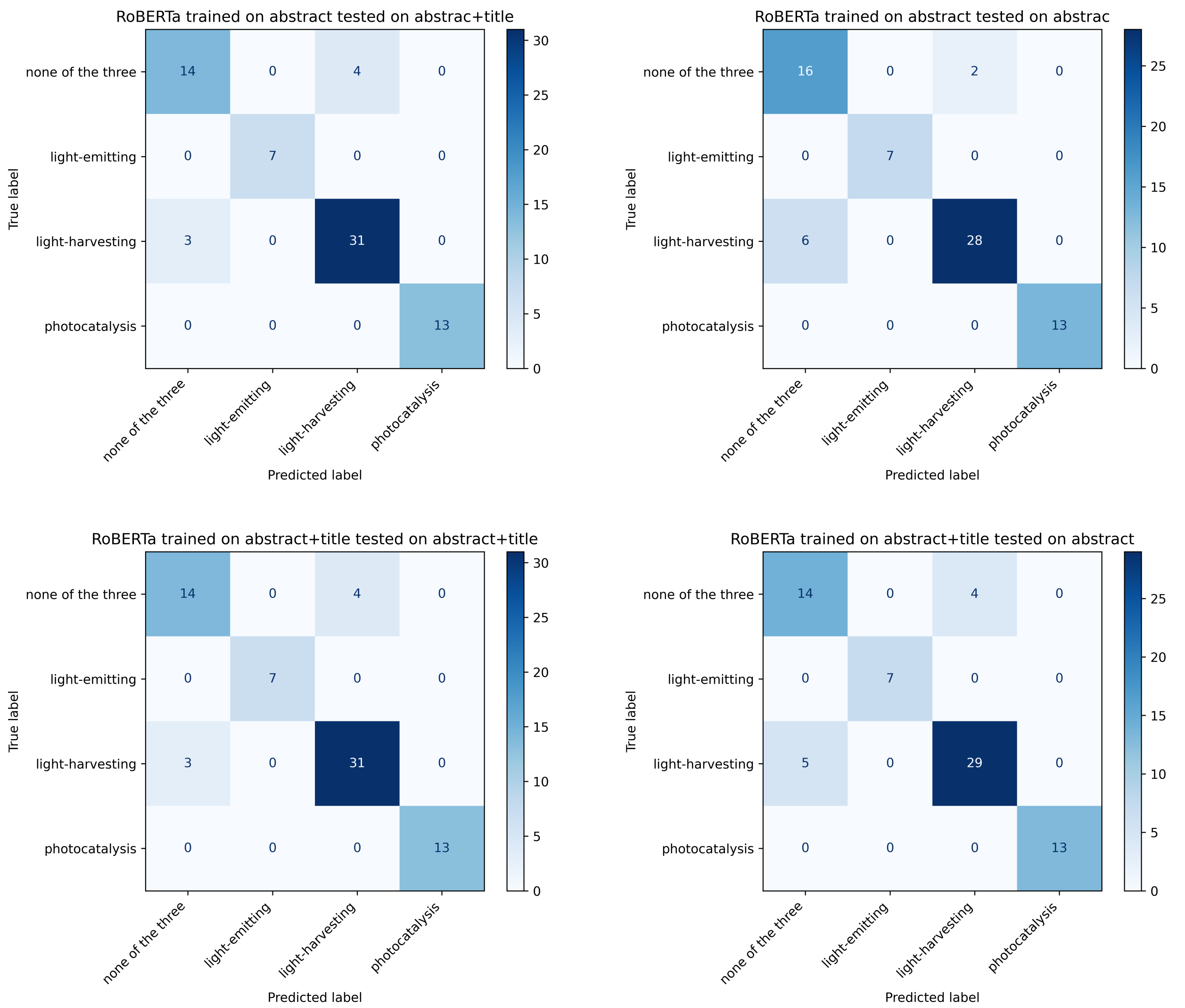

Figure S3: Confusion matrices of the results on the case study of classifying the 2023 top cited papers about optoelectronics, obtained with the OE-RoBERTa classification model under four different setups.
